# CD47 Binding on Vascular Endothelial Cells Inhibits IL-17-Mediated Leukocyte Adhesion

**DOI:** 10.3390/ijms23105705

**Published:** 2022-05-20

**Authors:** Laura Soriano-Romaní, Fayaz A. Mir, Niharika Singh, Ian Chin, Ali Hafezi-Moghadam, Sharmila Masli

**Affiliations:** 1 Ocular Surface Group, IOBA—University of Valladolid, Paseo de Belén 17, 47011 Valladolid, Spain; laurasorianoromani@gmail.com; 2 Department of Ophthalmology, Boston University School of Medicine, 72 East Concord Street, Boston, MA 02118, USA; fayaztrali@gmail.com (F.A.M.); nihsingh@bu.edu (N.S.); icgitar@gmail.com (I.C.); 3 Molecular Biomarkers Nano-Imaging Laboratory (MBNI), Brigham and Women’s Hospital and Department of Radiology, Harvard Medical School, 75 Francis Street, Boston, MA 02115, USA; ahm@bwh.harvard.edu

**Keywords:** CD47, chronic ocular inflammation, leukocyte adhesion, vascular endothelial cells, VCAM-1

## Abstract

To address the conflicting role of thrombospondin (TSP)-1 reported in acute and chronic pathologies, this study investigated the role of TSP-1 in regulating leukocyte recruitment and regulation of VCAM-1 expression using mouse models of uveitis. The spontaneously increased VCAM-1 expression and leukocyte adhesion in retinas of TSP-1-deficient mice suggested a TSP-1-mediated regulation of VCAM-1 expression. In a chronic uveitis model, induced by immunizing wild-type mice with specific interphotoreceptor retinoid-binding protein (IRBP) peptide, topically applied TSP-1-derived CD47-binding peptide significantly reduced the clinical disease course and retinal leukocyte adhesion as compared to the control peptide-treated group. In contrast, in LPS-mediated acute uveitis, TSP-1 deficiency significantly reduced the retinal leukocyte adhesion. The results of our in vitro study, using vascular endothelial cell (EC) cultures, demonstrate that unlike TNF-α, VCAM-1 expression induced by IL-17 is associated with a reduced expression of endogenous TSP-1. Such reduced endogenous TSP-1 expression in IL-17-stimulated ECs helps limit the CD36-mediated increased VCAM-1 expression, while favoring CD47-mediated inhibition of VCAM-1 expression and leukocyte adhesion. Thus, our study identifies TSP-1:CD47 interaction as a molecular pathway that modulates IL-17-mediated VCAM-1 expression, contributing to its anti-inflammatory effect in chronic inflammatory conditions.

## 1. Introduction

The expression of vascular cell adhesion molecule (VCAM)-1 in activated endothelial cells plays a significant role in adhesion of lymphocytes, monocytes, as well as neutrophils [1]. Such expression of VCAM-1 represents a downstream effect of pro-inflammatory cytokines that facilitates recruitment of leukocytes at the site of inflammation. Therefore, in addition to blocking cytokines such as TNF-α and IL-17, blockade of VCAM-1 ligand has also emerged as a successful therapeutic approach for autoimmune diseases [2]. Vascular endothelial cells are reported to respond to TNF-α by increasing their endogenous expression of TSP-1 to mediate increased expression of VCAM-1 through signals delivered via its receptor CD47 [3]. These results are considered consistent with a pro-inflammatory role of TSP-1, as concluded in several models of acute inflammation [4,5,6,7]. However, these studies fail to explain the observed spontaneous development of chronic autoimmune disease in TSP-1-deficient mice [8]. Clinical symptoms of this chronic pathology are improved, not only in response to VCAM-1 blockade [9], but also in response to CD47-binding agonist peptide derived from TSP-1 [10]. In fact, these reports support an anti-inflammatory role of TSP-1. The contrasting outcome of TSP1:CD47 interaction in acute vs. chronic inflammatory conditions suggests the existence of differential mechanisms mediated by TSP-1 under these conditions. 

In addition to CD47, vascular endothelial cells are known to express another TSP-1 receptor, CD36, known to deliver anti-angiogenic effect of TSP-1 by inducing apoptosis of endothelial cells [11,12]. However, it remains unknown if CD36-mediated signals can influence the outcome of TSP-1: CD47 interaction in endothelial cells. Considering that vascular endothelial cells endogenously express TSP-1 and increase its expression in response to cytokines such as TNF-α, it is more likely that TSP-1-mediated regulation of VCAM-1 expression involves ligation of both CD47 and CD36 receptors. It is possible that net signaling via both these receptors in vascular endothelial cells leads to contrasting TSP-1-mediated outcome in acute vs. chronic inflammatory conditions. 

Acute and chronic pathologies differ with respect to the predominance of inflammatory cytokines TNF-α vs. IL-17, respectively. Both these cytokines are reported to induce VCAM-1 expression on vascular endothelial cells [3,13,14]. During inflammation, early upregulation of VCAM-1 is largely attributed to TNF-α [15,16], and blockade of IL-17 is reported to reduce VCAM-1 expression in chronic inflammatory condition [17]. Thus, in addition to its angiogenic effect mediated by endothelial cell proliferation, IL-17 increases the expression of VCAM-1 on these cells to support inflammation by facilitating the extravasation of inflammatory effectors into the tissue [18]. While the contribution of TSP-1:CD47 signaling in TNF-α-mediated VCAM-1 expression has been addressed [3], it is not known if this signaling also regulates IL-17-mediated VCAM-1 expression. In this study, we examine both in vitro and in vivo effects of TSP-1 signaling through CD47 on IL-17-mediated VCAM-1 expression in vascular endothelial cells and the resulting changes in leukocyte adhesion. The anti-inflammatory role of TSP-1 in chronic pathology, as against acute inflammation, suggests that TSP-1 may deliver different signals in IL-17-stimulated vascular endothelial cells as compared to those stimulated by TNF-α. It is not known whether the anti-inflammatory effect of TSP-1 includes VCAM-1 regulation. 

In this study, we demonstrate the contrasting role of TSP-1 in acute vs. chronic models of experimental uveitis and examine the influence of CD47-mediated signaling on leukocyte adhesion in a chronic model of uveitis and its impact on the clinical disease course. We determine whether CD47 signaling modulates IL-17-induced VCAM-1 expression and leukocyte adhesion and address the contribution of CD36 to TSP-1-mediated changes in VCAM-1 expression on vascular endothelial cells. Together, our results in this study identify a potential mechanism that explains a conflicting role of TSP-1 in acute vs. chronic inflammatory conditions.

## 2. Results

### 2.1. Thrombospondin-1-Derived CD47-Binding Peptide Inhibits Vascular Leukocyte Adhesion in Chronic Uveitis

**Figure 1:**
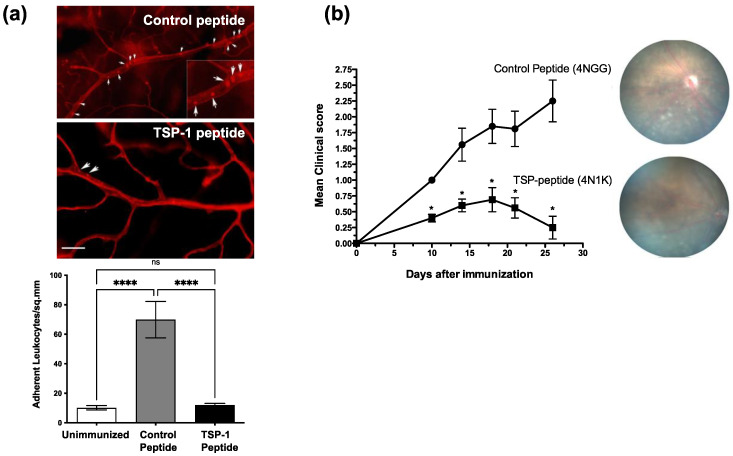
CD47-binding peptide inhibits leukocyte adhesion to retinal vessels in IRBP-induced chronic uveitis in mice. WT mice were immunized s.c. with IRBP (200 µg) in complete Freund’s Adjuvant and treated once daily with topical application of 4N1K or 4NGG (5 µg per eye). (**a**) Whole mounts of retinas were harvested 7 days post-immunization from rhodamine-ConA perfused mice and imaged to visualize firmly adherent leukocytes (arrows) in retinal vessels. The inset shows a digital magnification of an area. Quantitative analysis of adherent leukocytes is presented in the bar graph. Data are presented as the mean adherent leukocytes per mm^2^ ± SEM. (**b**) Clinical course of immunized and treated mice was followed for 30 days post-immunization by weekly fundus exam and scoring, as described in Materials and Methods. Results are expressed as the mean scores ± SEM with representative fundus images. *n* = 5 per group per experiment. * *p* < 0.05, **** *p* < 0.0001. Scale bar = 100 µM.

In an experimental model of chronic uveitis, IRBP immunization of WT mice induces increased expression of adhesion molecules on retinal vascular endothelium, which is followed by increased adherence of leukocytes [19]. We tested whether TSP-1-derived peptide can limit leukocyte adhesion in mice with uveitis and alter their disease course. We used CD47-binding peptide, 4N1K, and a control peptide, 4NGG. We immunized WT mice according to an established protocol for the induction of chronic uveitis [20]. Immunized mice were treated daily with topical application of 4N1K or 4NGG peptides (10 µg/mouse). In one experiment, we harvested retinal tissues after perfusing mice with rhodamine-conjugated Con A to visualize retinal vessels and adherent leukocytes, as described earlier. We detected significantly reduced leukocyte adhesion in mice treated with 4N1K as compared to those treated with 4NGG (Figure 1a). Moreover, these results also correlated with improved clinical symptoms, as observed over a period of 30 days post-immunization in 4N1K-treated mice compared to 4NGG-treated mice (Figure 1b). These results support a regulatory role of TSP-1 in leukocyte adhesion in chronic inflammatory conditions and is consistent with the spontaneous chronic inflammation reported in TSP-1-deficient mice [8,21]. 

### 2.2. The Lack of TSP-1 Prevents Retinal Leukocyte Adherence in Acute Uveitis Inflammation

**Figure 2:**
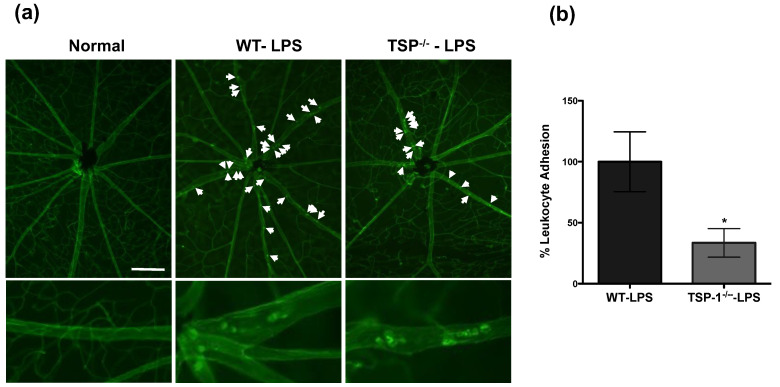
Expression of TSP-1 contributes to leukocyte adhesion to retinal vessels during LPS-induced acute uveitis inflammation in mice. Acute uveitis was induced in WT or TSP-1-deficient mice by administering 150 mg of LPS i.p. After 24 h, firmly adherent leukocytes in retinal vessels were visualized by perfusing the mice with rhodamine-conjugated ConA (red) before harvesting the retinas. (**a**) Representative images of retina whole mounts from untreated WT mice as normal and WT and TSP-1-deficient mice with EIU are marked with white arrows to indicate adherent leukocytes; (**b**) Quantitative assessment of adherent leukocytes in retinal vessels (*n* = 3–4 each group). Data are expressed as leukocyte adhesion relative to mean number of adherent leukocytes in WT retina ± SEM. * *p* < 0.05. Scale bar = 200 µM.

In contrast to our results in this study, a previous in vitro study reported that endogenous TSP-1 and its interaction with its receptor CD47 mediates increased expression of VCAM-1 in TNF-α-stimulated vascular endothelial cells [3]. We first sought to determine whether the apparent contradiction in our observation resulted from our in vivo approach. To address this issue, we used endotoxin-induced uveitis (EIU) model of acute ocular inflammation induced by administering lipopolysaccharide (LPS). In this model, TNF-α-mediated leukocyte adhesion in retinal vessels contributes to the pathogenesis [22,23]. We induced EIU in WT and TSP-1-deficient mice and evaluated leukocyte adhesion in retinal whole mounts 24 h after intraperitoneal (i.p) injection of LPS. As shown in Figure 2a, while increased numbers of adherent leukocytes were detected in LPS-injected WT retinas, very few leukocytes were detected in retinas from LPS-injected TSP-1-deficient mice. Quantitative assessment of adherent leukocytes indicated a significant reduction in leukocyte adhesion in TSP-1-deficient mice compared to WT mice (Figure 2b). These results support a possibility of TSP-1-mediated leukocyte adhesion during acute inflammation and are consistent with previously reported endothelial response to TNF-α. Thus, our in vivo studies reveal an opposing role of TSP-1 in acute and chronic inflammation. 

### 2.3. Increased VCAM-1 Expression and Leukocyte Adhesion in Retinal Vessels of TSP-1-Deficient Mice

**Figure 3:**
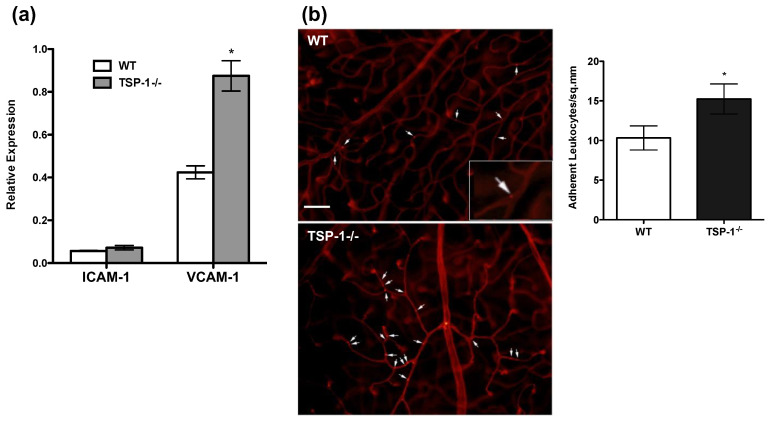
The expression of VCAM-1 and adherence of leukocytes is increased in TSP-1-deficient blood vessels in the retina. (**a**) Real-time PCR was performed on RNA isolated from retinas harvested from WT or TSP-1-deficient (TSP-1^−/−^) mice (8 weeks old, *n* = 5 each group) to detect message levels for ICAM-1 and VCAM-1 genes. Results are expressed as the mean ± SEM. (**b**) Firmly adherent leukocytes in retinal vessels were visualized by perfusing mice with rhodamine-conjugated Con A (red) before harvesting retina. Representative images of retina whole mounts from WT and TSP-1-deficient mice are shown with a quantitative assessment of adherent leukocytes in retinal vessels (*n* = 4 each group). White arrows indicate adherent leukocytes in the main images, and the inset shows a digital magnification of an area. Data are expressed as the mean number of leukocytes per mm^2^ ± SEM. * *p* < 0.05. Scale bar = 75 µM.

Earlier studies have demonstrated that TSP-1 deficiency in mice results in development of an exacerbated chronic uveitis inflammation following IRBP immunization as compared to that seen in similarly immunized wild-type (WT) control mice [20]. While no overt retinal inflammation is detectable in unimmunized TSP-1-deficient mice, previous studies did not evaluate the expression of adhesion molecules in retinal vessels. A comparison of the expression of ICAM-1 and VCAM-1 in retinal tissues harvested from TSP-1-deficient and WT mice in a real-time PCR revealed significantly increased expression of VCAM-1 in TSP-1-deficient retina compared to WT controls (Figure 3a). There was no such difference detectable in the message level for ICAM-1. These results were also confirmed by immunostaining (data not shown). To determine whether increased VCAM-1 expression in the retinal vessels translates to increased leukocyte adhesion, we perfused WT and TSP-1-deficient mice with rhodamine-conjugated Concanavalin A (Con A) before harvesting retinal tissues to enable visualization and enumeration of intravascular leukocyte adhesion. As expected, significantly increased numbers of adherent leukocytes were detected in retinal tissues harvested from TSP-1-deficient mice compared to WT controls (Figure 3b). Together, these results suggest a role of TSP-1 in inhibiting VCAM-1 expression on vascular endothelial cells. 

### 2.4. IL-17 Induces VCAM-1 Expression in Endothelial Cells Independently of Their Endogenous TSP-1 

**Figure 4:**
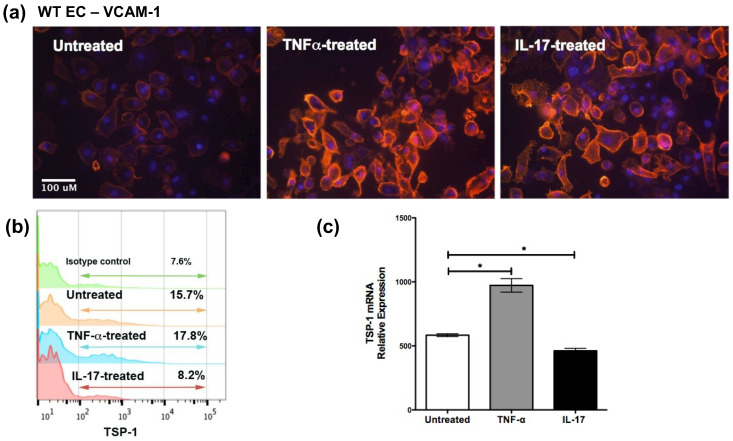
IL-17 and TNF-α both increase expression of VCAM-1 on vascular endothelial cells but differentially modulate endogenous TSP-1 expression. Primary cultures of murine vascular endothelial cells were treated with TNF-α (10 ng/mL) or IL-17 (10 ng/mL) for 24 h. (**a**) Endothelial cells were immunostained with anti-VCAM-1 (red), as described in Materials and Methods (200× magnification), nuclei stained with DAPI (blue) (**b**) Intracellular TSP-1 in permeabilized endothelial cells was detected by flow cytometry. Percentage indicates the proportion of cells positively stained for TSP-1, (**c**) Real-time PCR was performed on RNA isolated from endothelial cells to detect TSP-1 message. Results are expressed as the mean ± SEM (*n* = 3 per experiment; * *p* < 0.05).

Considering that IL-17 was demonstrated to play a dominant role in IRBP-induced model of chronic uveitis [24], next, we confirmed that the expression of VCAM-1 on primary mouse vascular endothelial cells is induced by IL-17, as previously reported in human vascular endothelial cells (HUVEC) [13]. Similar to TNF-α, IL-17 induced VCAM-1 expression in mouse vascular endothelial cells cultured in the presence of TNF-α or IL-17 (10 ng/mL) (Figure 4a). We then compared the endogenous expression of TSP-1 in untreated, TNF-α- and IL-17-treated mouse endothelial cells. The mean fluorescence intensity of TSP-1 staining determined using ImageJ analysis indicated significantly reduced staining in IL-17-treated cells compared to TNF-α-treated cells (15.5 ± 2.8 vs. 26.3 ± 4.5, respectively, *p* < 0.05, *n* = 16). Quantitative analysis by flow cytometry consistently revealed increased proportion of cells stained positively for intracellular TSP-1 after TNF-α treatment as compared to untreated cells (Figure 4b). However, IL-17 treatment of endothelial cells resulted in nearly 50% reduction in the number of TSP-1-positive cells. This observation was confirmed by significantly increased TSP-1 message levels in TNF-α and reduced levels in IL-17-treated cells compared to untreated cells (Figure 4c). Together, these results suggest that unlike TNF-α, IL-17-mediated VCAM-1 expression in vascular endothelial cells may not be mediated by their endogenous expression of TSP-1.

### 2.5. IL-17-Induced VCAM-1 Expression on Vascular Endothelial Cells and Leukocyte Adhesion Are Inhibited by CD47 Ligation

**Figure 5:**
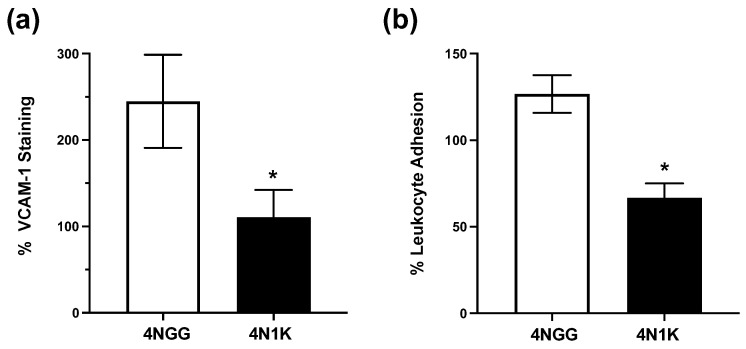
CD47-mediated signals in vascular endothelial cells inhibit IL-17-induced VCAM-1 expression and leukocyte adhesion. Primary cultures of vascular endothelial cells from WT mice were treated overnight with IL-17 (10 ng/mL) alone or in the presence of CD47-binding 4N1K or control 4NGG peptides (10 nM). (**a**) To determine VCAM-1 expression, cells immunostained for VCAM-1 (red) (200×) were imaged and analyzed using ImageJ software, as described in Materials and Methods. Nuclei were stained with DAPI (blue). Results are shown as the mean fluorescence staining relative to untreated cells ± SEM; (**b**) To determine leukocyte adhesion, treated cells were washed gently and overlaid with CFSE-labeled leukocytes, as described in Materials and Methods, and after washing away non-adherent cells, fluorescence intensity of adherent cells was measured using a fluorescence microplate reader. Data are presented as the mean fluorescence intensity of adherent cells relative to untreated cells ± SEM. *n* = 3–4 each treatment. * *p* < 0.05.

To determine whether the observed inhibitory effect of CD47-binding TSP-1 peptide on retinal leukocyte adhesion in our in vivo experiments included a direct effect on IL-17-stimulated vascular endothelial cells during chronic uveitis, we conducted in vitro experiments. Vascular endothelial cells harvested from WT mice were treated with IL-17 in the presence of 10 nM 4N1K or 4NGG (control peptide). As shown in Figure 5a, significantly reduced VCAM-1 expression was detected in cells treated with 4N1K peptide compared to controls (representative images in Supplementary Figure S1). Further, we also tested whether this change in VCAM-1 expression correlated with the reduced leukocyte adhesion. To address this possibility, we performed leukocyte–endothelial cell adhesion assay using CFSE-labeled leukocytes, as described in Materials and Methods. At the completion of this assay, significantly reduced fluorescence intensity was detected, corresponding to reduced numbers of adherent leukocytes in endothelial cells treated with IL-17 and 4N1K as compared to cells treated with IL-17 and 4NGG (Figure 5b). These results support a direct inhibitory effect of CD47 binding on IL-17-mediated VCAM-1 expression and related leukocyte adhesion to vascular endothelial cells. These results are consistent with our findings in the chronic uveitis model.

### 2.6. Endogenous TSP-1 Expressed by Vascular Endothelial Cells Mediates Increased VCAM-1 Expression via CD36 Binding 

Our observation that TSP-1: CD47 interaction downregulates VCAM-1 expression in IL-17-stimulated vascular endothelial cells may appear in direct contrast to previously reported observation that the same interaction increases VCAM-1 expression in TNF-α-stimulated cells [3]. However, previously, the role of CD36 expressed on vascular endothelial cells was not considered in interpreting the outcome of TSP-1-driven increased VCAM-1 expression. To resolve the apparent dichotomy, we addressed the potential contribution of TSP-1: CD36 interaction in regulating VCAM-1 expression on vascular endothelial cells. We hypothesize that the increased endogenous TSP-1 (as induced by TNF-α but not IL-17) favors TSP-1: CD36 interaction to mediate increased VCAM-1 expression.

**Figure 6:**
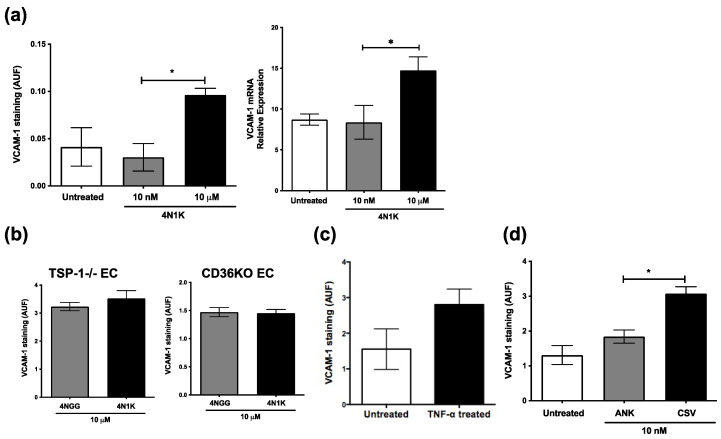
Endogenous TSP-1 from vascular endothelial cells induces increased VCAM-1 expression via CD36. Primary vascular endothelial cell cultures were treated overnight with 10 nM or 10 mM of CD47-binding 4N1K or control peptide 4NGG (*n* = 4 per group). (**a**) Peptide-treated WT cells were immunostained for VCAM-1, imaged at 200×, and fluorescence intensities (AUF) were analyzed using ImageJ, and their RNA was analyzed in real-time PCR to detect VCAM-1 message level relative to housekeeping gene GAPDH (*n* = 3 reactions per group); (**b**–**d**) Fluorescence intensity of VCAM-1 immunostaining in endothelial cells (EC) derived from TSP-1- or CD36-deficient mice treated with indicated peptides (**b**); in untreated or TNF-α-treated TSP-1-deficient endothelial cells (**c**) and in WT endothelial cells treated with CD36-binding peptide CSV or control peptide ANK (**d**). Data are presented as the mean values ± SEM. * *p* < 0.05. Together, these results suggest that VCAM-1 expression on vascular endothelial cells is differentially regulated by two receptors of TSP-1. While increased endogenous TSP-1 expression favors CD36-mediated signaling causing increased VCAM-1 expression, reduced endogenous TSP-1 expression allows for CD47-mediated downregulation of VCAM-1. These results not only explain the dichotomy in our results from those reported previously but also shed light on the opposite roles of TSP-1 in acute vs. chronic inflammation with predominant roles for TNF-α vs. IL-17, respectively.

To address this hypothesis, first, we determined whether CD47-binding TSP-1 peptide (4N1K) could mimic previously reported endothelial cell response to TNF-α. We evaluated VCAM-1 expression in WT endothelial cells exposed to 10 nM and 10 µM of 4N1K. Our results demonstrate an increased VCAM-1 expression in response to higher (10 µM) concentration of 4N1K as compared to lower concentration (10 nM) or untreated controls, both by immunostaining as well as real-time PCR (Figure 6a). We confirmed the engagement of CD47 in this experiment, as no increase in VCAM-1 expression was detected in the presence of CD47-blocking antibody (Supplementary Figure S2a). Moreover, endothelial cells treated with 10 µM 4N1K increased their expression of endogenous TSP-1 (Supplementary Figure S2b). However, such peptide treatment failed to increase VCAM-1 expression in endothelial cells derived from TSP-1- or CD36-deficient mice (Figure 6b). Similarly, CD36-deficient vascular endothelial cells failed to increase VCAM-1 expression in response to TNF-α stimulation (Figure 6c). Furthermore, a CD36-binding TSP-1 peptide (CSV) induced significantly increased VCAM-1 expression compared to control peptide (ANK) in WT endothelial cells (Figure 6d). Together, these results clearly demonstrate the involvement of CD36 in driving TSP-1-mediated increased VCAM-1 expression. Therefore, it is possible that increased endogenous TSP-1 in TNF-α-stimulated endothelial cells permits dominance of CD36 signaling to mediate increased VCAM-1 expression. However, in IL-17-stimulated cells, a reduced endogenous TSP-1 allows inhibitory signaling via CD47 to dominate and downregulate VCAM-1 expression. 

## 3. Discussion

Contrary to a pro-inflammatory role attributed to TSP-1 by some reports, we provide evidence in this study that further supports an anti-inflammatory role of TSP-1. Other investigators have reported that TSP-1: CD47 interaction in endothelial cells mediates TNF-α-driven VCAM-1 expression associated with acute inflammation [3]. Our results in this study, however, demonstrate that TSP-1: CD47 interaction in vascular endothelial cells inhibits IL-17-induced VCAM-1 expression associated with chronic inflammation. Such inhibitory effect detected in vitro also translates to functional inhibition of leukocyte adhesion to endothelial cells, both in vitro and in vivo. These observations further correlate with significantly improved clinical course of chronic uveitis in a mouse model where IL-17 dominates as an inflammatory effector [24]. Our in vivo results confirm TSP-1-dependent leukocyte adhesion during TNF-α-mediated acute inflammation, and the in vitro results clarify that increased endogenous TSP-1 induced by TNF-α favors pro-inflammatory signaling via CD36 to increase VCAM-1 expression. Failure to induce such increased endogenous TSP-1 by IL-17, on the other hand, allows for the anti-inflammatory CD47-mediated signaling that downregulates VCAM-1 expression. Thus, TSP-1 clearly exerts differential effects on VCAM-1 expression in vascular endothelial cells via their CD47 and CD36 receptors. These results now offer a potential explanation of the paradoxical effects of TSP-1 often reported in the context of inflammation.

**Figure 7:**
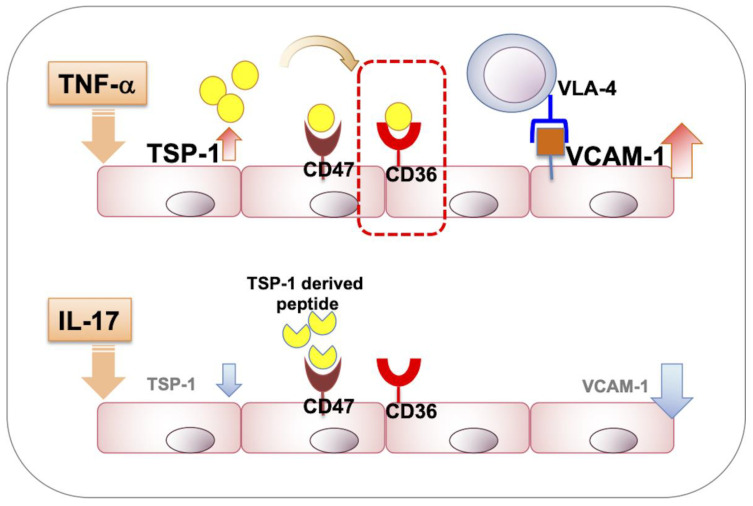
Schematic representation of the involvement of TSP-1 and its receptors, CD47 and CD36, in regulating VCAM-1 expression in vascular endothelial cells exposed to TNF-α vs. IL-17. Predominant signaling via CD36 drives increased VCAM-1 expression mediated by endogenous TSP-1, which is increased in response to TNF-α. However, IL-17 exposure of cells reduces their endogenous TSP-1 expression, allowing the dominance of inhibitory CD47 signaling that downregulates VCAM-1 expression.

Chronic autoimmune pathologies have been predominantly associated with IL-17 [25], whereas TNF-α is secreted in response to acute inflammation [22,26,27]. Neutralizing these cytokines with antibodies in chronic and acute inflammation models, respectively, results in reduced expression of vascular endothelial VCAM-1 [15,17]. Recent studies related to chronic uveitis have reported a predominance of CD4+ T cells among inflammatory infiltrates in the retina and a particularly predominant contribution of IL-17 producing CD4+ T cell subset (Th17) to the clinical disease course in IRBP-immunized EAU mouse model [28,29]. Consistent with these reports, our results demonstrate increased VCAM-1 expression in vascular endothelial cells in response to IL-17, similar to that induced by TNF-α. Despite their similar effects on VCAM-1 expression, TNF-α and IL-17 clearly differ in their ability to regulate endogenous TSP-1 expression in vascular endothelial cells, with the former increasing and the latter reducing it. Our flow cytometry analysis also reveals that change in TSP-1 expression is restricted to a subset of vascular endothelial cells likely derived from post-capillary venules. This observation is consistent with the leukocyte trafficking function of endothelial cells, almost exclusively associated with this subset of micro-vessels [30,31]. Overall, our studies indicate that chronic inflammation and IL-17 are associated with reduced TSP-1 expression, in contrast to its increased expression reported in several models of acute inflammation [4,5,6,7]. Considering that at higher TSP-1 concentrations, the CD36 signaling is favored [32], it is conceivable that in acute inflammatory conditions, TNF-α-mediated increased TSP-1 results in dominant CD36 signaling. Reduced TSP-1 levels associated with chronic inflammation involving IL-17, on the other hand, may allow CD47-mediated inhibitory signaling (Figure 7). Thus, the net signaling from CD36 and CD47 in endothelial cells is affected by their endogenously produced TSP-1, which is differentially regulated during acute vs. chronic inflammation.

The possibility that seemingly contradictory effects of TSP-1 actually result from signaling via two different receptors is supported collectively by in vitro and in vivo evidence in this study. We demonstrate in vitro that, in the absence of increased endogenous TSP-1, the nanomolar concentration of CD47-binding peptide reduces IL-17-mediated VCAM-1 expression and leukocyte adhesion to cultured vascular endothelial cells. These results are consistent with an earlier finding, which demonstrated that unlike the CD36 receptor, CD47 signaling can be induced by low (picomolar) concentrations of TSP-1 [32]. However, our results now clarify that TNF-α-induced VCAM-1 expression requires the expression of both TSP-1 and CD36 by endothelial cells. Thus, in acute inflammation, TSP-1 appears to be essential for facilitating endothelial adhesion of inflammatory leukocytes, and this was evident in our in vivo study. We noted a significant reduction in retinal leukocyte adhesion in LPS-induced acute uveitis in TSP-1-deficient mice. A similar effect on leukocyte adhesion was also reported by others after TNF-α blockade in LPS-induced uveitis in normal mice [22]. Together, these results underscore a differential role of TSP-1 in acute vs. chronic inflammatory processes. 

Previously, we have reported that topically administered CD47-binding peptide, 4N1K, ameliorates chronic ocular inflammation that develops spontaneously in TSP-1-deficient mice [10]. Studies reported by us and others have identified anti-inflammatory effect of CD47 ligation on T cells and antigen-presenting cells [10,33,34]. In our studies, we also confirmed the specificity of 4N1K signaling via CD47 to rule out any non-specific interactions of the peptide. Therefore, it may be argued that in the present study, the inhibition of retinal vascular leukocyte adhesion in 4N1K-treated mice with uveitis represents a secondary effect of dampened inflammatory immune effectors. However, systemic change, such as an induction of a regulatory immune response, was detected after two weeks of topical application of 4N1K peptide. In contrast, inhibition of leukocyte adhesion reported in this study was detected within 7–8 days of peptide treatment. It has been reported that topically administered drugs reach the posterior segment of the eye most likely via the conjunctival-scleral route with an extremely low bioavailability in the range of 0.0001–0.0004% [35]. Therefore, in our experiments, it is possible that nanomolar to picomolar concentrations of the topically applied 4N1K (725 µM) reach retinal vascular endothelial cells, exerting a direct anti-inflammatory effect. Further study documenting such bioavailability of CD47 peptide in retinal vessels is warranted. Such information can provide a valuable non-invasive therapeutic approach to address retinal inflammation where accessibility of retinal tissue presents major limitations for current treatments. 

In conclusion, our study identifies TSP-1: CD47 interaction mediated the regulation of IL-17-induced VCAM-1 expression. Our results help delineate the pro-inflammatory and anti-inflammatory effects of TSP-1 in the context of acute vs. chronic inflammation, respectively. In response to acute inflammation, vascular endothelial cells increase their endogenous TSP-1 expression, allowing the dominance of CD36-mediated pro-inflammatory signaling over the inhibitory CD47-mediated anti-inflammatory signaling. The latter is evident in chronic inflammation in the absence of increased endogenous TSP-1. Overall, this study also supports CD47 ligation as a promising therapeutic alternative to blocking adhesion molecules in chronic inflammatory diseases.

## 4. Materials and Methods

### 4.1. Mice

C57BL/6 (H-2b) male mice, 4 to 12 weeks of age, were purchased from Charles River Laboratories (Wilmington, MA, USA). A breeding pair of TSP-1-deficient mice (C57BL/6 background) was purchased from Jackson Laboratories (Bar Harbor, Maine, USA). A breeding pair of CD36KO mice (C57BL/6 background) was received from Dr. M. Freeman (Massachusetts General Hospital, Harvard Medical School, Boston, MA, USA). These mice were subsequently bred in-house in a pathogen-free facility at Boston University School of Medicine, Boston, MA, USA. 

The Institutional Animal Care and Use Committee at Boston University School of Medicine, Boston, approved animal studies described in this manuscript in accordance with the NIH guide for the care and use of laboratory animals (AN-15400). Some initial uveitis experiments were conducted while the Masli laboratory was at Schepens Eye Research Institute in Boston, prior to moving to Boston University in accordance with the institutional guidelines. The collaboration with the Hafezi-Moghadam laboratory started prior to this lab’s move from the Massachusetts Eye and Ear Infirmary to the Brigham and Women’s Hospital. All animal experiments were conducted in accordance with the ARVO Statement for the Use of Animals in Ophthalmic and Vision Research.

### 4.2. Antibodies and Reagents

Antibodies—anti-VCAM-1 (Biolegend, San Diego, CA, USA), anti-TSP-1 (Santa Cruz Biotechnology, Santa Cruz, CA, USA), anti-VE-cadherin and fluorochrome-conjugated anti-CD45 and anti-CD31 (BD Biosciences, San Jose, CA, USA).

Recombinant proteins—mouse IL-17 and TNF-α (R&D Systems, Minneapolis, MN, USA). Type 1A collagenase (Sigma-Aldrich St. Louis, MO, USA) and Type I collagenase (Worthington Biochemical Corp., Lakewood, NJ, USA). 

Peptides—TSP-1-derived peptides 4N1K (KRFYVVMWKK, CD47-binding peptide) and CSV (CSVTCG, CD36-binding peptide), and control peptides 4NGG (KRFYGGMWKK) and ANK (ANKHYF), respectively (Bio Basic, Markham, Ontario, Canada). 

Culture medium—DMEM containing 10 mM HEPES, 0.1 mM NEAA, 1 mM sodium pyruvate, 100 U/mL penicillin, 100 mg/mL streptomycin, 200 mM L-glutamine (Lonza, Basel, Switzerland), 10% fetal bovine serum (Atlanta Biologicals, Lawrenceville, GA, USA) and endothelial growth supplements (Cell Biologics Inc., Chicago, IL, USA), all purchased from Sigma–Aldrich, unless otherwise stated.

### 4.3. Primary Cultures of Murine Vascular Endothelial Cells

Murine vascular endothelial cells were obtained from WT and CD36KO mice. Briefly, collagenase digestion of lungs was performed by intratracheal instillation of type IA collagenase prior to harvesting lungs and subsequent incubation with type 1 collagenase at 37 °C for 45 min. The digested tissue was filtered through a 70 μm mesh, and dissociated cells were cultured in gelatin-coated tissue culture flasks at 37 °C with 5% CO_2_ for a period of up to 7 days. Confluent cultures were trypsinized, and cells were stained with fluorochrome-conjugated CD45, CD31 and VE-cadherin antibodies for flow cytometric sorting (MoFlo Legacy, Boston University Flow Cytometry Core Facility). Cells negatively stained for CD45 and positively stained for endothelial markers VE-cadherin/CD31 were sorted as endothelial cells and used in experiments at indicated numbers. 

### 4.4. Immunostaining and Analysis

For microscopy, primary cultures of murine vascular endothelial cells were seeded onto fibronectin-coated 8-well chamber slides or 96-well plates with or without cytokines and/or TSP-1-derived peptides, depending on the experimental setup, and cultured overnight. Cells were fixed with cold methanol (for TSP-1 staining) or paraformaldehyde (for VCAM-1 staining) for 10 min and blocked with phosphate-buffered saline (PBS) containing 2% bovine serum albumin at room temperature (RT) for 1 h. For TSP-1 staining, a previous step to allow cell permeabilization was required using 0.3% Triton X-100 in PBS for 15 min. Thereafter, cells were rinsed with PBS and incubated with primary antibodies against VCAM-1 or TSP-1 in blocking buffer at RT for 2 h. Following incubation, cells were rinsed with PBS and incubated with fluorochrome-conjugated secondary antibodies at RT for 1 h. Nuclei were counterstained using 4′, 6-diamidino-2-phenylindole (DAPI). Negative controls were stained similarly, with omission of the primary antibody. Cell staining was evaluated using a fluorescence microscope (Olympus FSX100, Tokyo, Japan) using the same exposure time, gain and intensity. Semiquantitative analysis of staining intensities was performed as previously described [36]. Briefly, two-channel micrographs were analyzed using ImageJ software (http://imagej.nih.gov/ij/; National Institutes of Health, Bethesda, MD, USA, accessed in December 2014 and May 2018) to determine the thresholds in each channel and mean gray value in each corresponding channel. The results, in arbitrary units, were calculated as the ratio of the mean gray value for the color channel, corresponding to the protein of interest, to that obtained from the blue channel, corresponding to the DAPI-stained nuclei. Five to six images taken from different areas were analyzed in each group per experiment. Mean fluorescence intensity (MFI) of staining was calculated as mean fluorescence units of the staining per DAPI positive cell. Images containing equivalent cell numbers were used to determine MFI.

For flow cytometry, endothelial cells were stained with fixable viability dye eFluor^TM^ 780 (eBioscience, San Diego, CA, USA), fixed and permeabilized followed by incubation with anti-TSP-1 and fluorochrome-conjugated secondary antibody. Cell staining was analyzed using BD LSRII Flow Cytometer (Boston University Flow Cytometry Core Facility, 650 Albany street, Boston, MA 02118, USA). Further analysis of the data was performed using FlowJo v9.4.10 software (Tree Star, Inc., Ashland, OR, USA).

### 4.5. Leukocyte–Endothelial Cell Adhesion Assay

Leukocytes (lymph node cells) were labeled with 1 µM of 5(6)-carboxyfluorescein N-hydroxysuccinimidyl ester (CFSE) at RT for 8 min followed by thorough washing to remove excess dye. Labeled cells were then incubated with confluent endothelial cells cultures in a 96-well plate (1 × 10^5^ cells/well) at 37 °C for 30 min. After incubation, cultures were washed 3 times with PBS to remove non-adherent cells, and the fluorescence of adherent cells was measured using Synergy H1 microplate reader (Biotek, Winooski, VT, USA) at 485 nm/530 nm. The fluorescence intensity in each well correlated with the number of adherent leukocytes. Each experimental sample was set up in triplicates, and the results were calculated as mean fluorescence intensity. 

### 4.6. Experimental Autoimmune Uveitis (EAU) Model—Peptide Treatment and Disease Monitoring

Induction of EAU was achieved through immunizing C57Bl/6 mice with human interphotoreceptor retinoid binding protein (IRBP) peptide 1–20 (GPTHLFQPSLVLDMAKVLLD, 10 mg/mL; Sigma Genosys, Cambridge, UK) emulsified 1:1 in complete Freund’s adjuvant (CFA) (Difco Laboratories, Detroit, MI, USA). Each mouse received s.c. injection of 100 μg IRBP and i.p. injection of 0.1 μg of Bordetella pertussis toxin (Health Protection Agency, Salisbury, UK). Mice were treated topically with 4N1K peptide or control peptide (10 μg/mouse) bilaterally (5 µL/eye), once a day for 1 week post-immunization. Retinas were examined at regular intervals throughout a 30-day period. The extent of uveitis is represented as the mean EAU score for both eyes on the day of examination. Disease severity was clinically assessed by ocular fundus examinations performed using slit lamp in animals under anesthesia. Pupils were dilated with 1% tropicamide ophthalmic solution (Akorn, Buffalo Grove, IL, USA) before examination. Clinical scoring of EAU was based on the number of white, focal, perivascular lesions and the extent of retinal vessel exudates, hemorrhage and detachment. Clinical severity was graded on a 0 to 5 scale, as described previously [20]. Fundus images were taken using the Micron III Retinal Imaging Microscope (Phoenix Research Laboratories, Pleasanton, CA, USA) at Schepens Eye Research Institute, Boston, MA.

### 4.7. Quantification of Adherent Leukocytes in Retinal Vessels

Unimmunized and IRBP-immunized mice (day 9 post-immunization) treated with either control or 4N1K peptide were anesthetized and perfused with 10 mL of PBS to remove intravascular content, including non-adherent leukocytes. Perfusion with 10 mL of Rhodamine-conjugated Concanavalin A (ConA, 5 µg/mL in PBS pH 7.4) (Vector labs, Burlingame, CA, USA) was performed to label adherent leukocytes and vascular endothelial cells. Residual unbound lectin was removed by perfusing an additional 10 mL of PBS. The carefully harvested retinas were flat mounted in a mounting medium (Vector labs). Each retina was imaged with fluorescence microscope (Olympus FSX100, Tokyo, Japan), and the adherent leukocytes per retina were counted. Similar assessments were performed on wild-type and TSP-1-deficient mice 24 h after injecting 100 µg of LPS from Salmonella typhimurium (Sigma Chemical, St. Louis, MO, USA) in one hind footpad.

### 4.8. Real-Time PCR

Total RNA was isolated from the treated primary cultures of vascular endothelial cells and from WT or TSP-1-deficient mouse retinas using TRIzol Reagent (Life Technologies, Carlsbad, CA, USA) according to the manufacturer’s instructions. cDNA was synthesized using the SuperScript VILO cDNA kit (Life Technologies). Real-time PCR was performed on a 7200 Real Time System (Applied Biosystems, Carlsbad, CA, USA) using SYBR Green PCR Master Mix (Life Technologies) to determine relative quantitative expression levels of VCAM-1, intercellular adhesion molecule (ICAM)-1 and TSP-1. VCAM-1 primers (F-5′-CCCGAAACATGGATAATCCT-3′ and R-5′-ATTGTGAGCCAACTTCAGTCTTAG-3′), ICAM-1 primers (F-5’-AGACGCAGAGGACCTTAACAGTC-3’ and R-5’-GGGCTTCACACTTCACAGTTACTT-3’), TSP-1 primers (F-5′-AAGAGGACCGGGCTCAACTCTACA-3′ and R-5′-CTCCGCGCTCTCCATCTTATCAC-3′) and glyceraldehyde-3-phosphate dehydrogenase (GAPDH) primers (F-5′-CGAGAATGGGAAGCTTGTCA-3′ and R-5′-AGACACCAGTAGACTCCACGACAT-3′) were used. The amplification reactions were set up in quadruplicates with the following thermal profile: 95 °C for three min, 40 cycles at 95 °C for 20 s, 53 °C for 30 s and 72 °C for 40 s. To verify the specificity of the amplification reaction, a melting curve analysis was performed. The fluorescence signal generated at each cycle was analyzed using system software. The threshold cycle values were used to determine relative quantification of gene expression with GAPDH as a reference gene.

### 4.9. Statistical Analysis

Normal distribution of the data was assessed using the Shapiro–Wilk test. Significant differences between mean values of the experimental and control groups were determined using Student’s *t*-test for normally distributed data. In the absence of normal distribution, a Kruskal–Wallis test with Dunn’s test to correct for multiple comparisons was used to compare multiple groups, and Mann–Whitney U-test was used to compare clinical disease course between the two groups. Data were expressed as the mean ± standard error of the mean (SEM). *p* < 0.05 was considered statistically significant.

## Supplementary Materials

The following are available online at https://www.mdpi.com/article/10.3390/ijms23105705/s1.
