# Supplementary material for: CD47 Binding on Vascular Endothelial Cells Inhibits IL-17-Mediated Leukocyte Adhesion
Source: Int J Mol Sci. 2022 May 20;23(10):5705. doi: 10.3390/ijms23105705 (PMC9146020; doi:10.3390/ijms23105705)
Supplement: Supplementary file 1 [file ijms-23-05705-s001.zip › ijms-1717550 Supplementary.pdf]

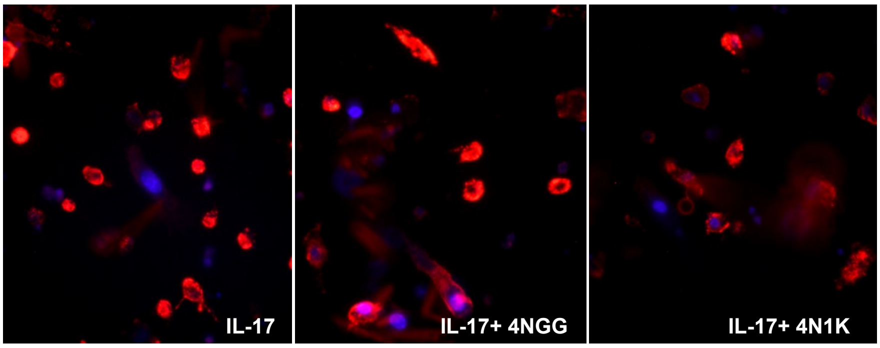

Figure S1. Immunostaining of VCAM-1 in WT endothelial cells treated with IL-17 and TSP-1 peptide. Primary cultures of vascular endothelial cells from WT mice were treated overnight with IL-17 (10 ng/mL) alone or in the presence of CD47 binding 4N1K or control 4NGG peptides (10 nM). Cells were immunostained for VCAM-1 (red) and nuclei were stained with DAPI (blue). Representative images show VCAM-1 immunostaining in ECs treated with IL-17 and 4N1K is reduced compared to controls treated with IL-17 alone or with control peptide 4NGG (200x magnification). .

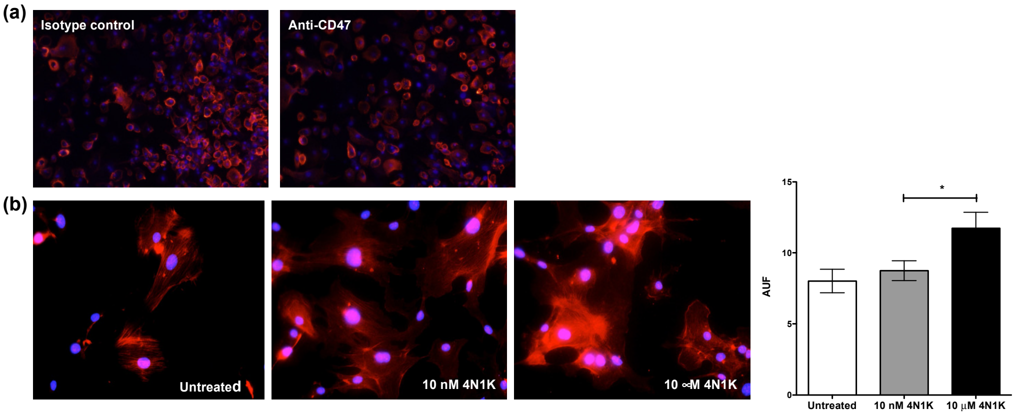

Figure S2. Immunostaining of VCAM-1 and TSP-1 in WT endothelial cells. (a) Representative images showing comparable MFI of VCAM-1 immunostaining in ECs treated with 10 µM 4N1K in the presence of CD47-blocking antibody as compared to the isotype control (0.59 vs. 0.69, n=12) indicates that VCAM-1 expression is not increased when CD47 signaling is blocked (100x magnification) ; (b) Representative images showing increased TSP-1 immunostaining in ECs treated with 10 µM concentration of CD47-binding peptide 4N1K as compared to untreated and 10nM peptide treated cells (200x magnification). * $p<0.05$.
